# Perturbation of the T cell receptor repertoire occurs with increasing age in dogs

**DOI:** 10.1016/j.dci.2017.10.020

**Published:** 2018-02

**Authors:** Angela Holder, Samantha M. Mirczuk, Robert C. Fowkes, Donald B. Palmer, Richard Aspinall, Brian Catchpole

**Affiliations:** a Department of Pathobiology and Population Sciences, Royal Veterinary College, North Mymms, Hertfordshire, UK; b Department of Comparative Biomedical Sciences, Royal Veterinary College, London, UK; c Health and Wellbeing Academy, Postgraduate Medical Institute, Anglia Ruskin University, Chelmsford, Essex, UK

**Keywords:** Immunosenescence, T cell receptor repertoire, CDR3 spectratyping, Aging, Canine

## Abstract

Immunosenescence is the gradual deterioration in immune system function associated with ageing. This decline is partly due to involution of the thymus, which leads to a reduction in the output of naive T cells into the circulating lymphocyte pool. Expansion of existing naive and memory T cell populations, to compensate for the reduction in thymic output, can lead to reduced diversity in the T cell repertoire with increasing age, resulting in impairment of immune responses to novel antigenic challenges, such as during infection and vaccination. Since associations between T cell repertoire and age have only been examined in a limited number of species, to gain further insights into this relationship, we have investigated age-related changes in the canine T cell receptor (TCR) repertoire.

Blood samples were obtained from Labrador retriever dogs of varying ages and variation in the complementary determining region 3 (CDR3) of the T cell receptor beta (TCRB) chain was investigated. CDR3 size spectratyping was employed to evaluate clonal expansion/deletion in the T cell repertoire, allowing identification of profiles within individual variable (V) region families that skewed away from a Gaussian distribution. Older dogs (10–13 years) were found to have an increased number of *TCRB* V gene spectratypes that demonstrated a skewed distribution, compared with young dogs (≤3 years). Additionally, there was a reduction in the number of clonal peaks present in the spectratypes of old dogs, compared with those of young dogs. The study findings suggest that there is an age-associated disturbance in the diversity of the T cell receptor repertoire in dogs.

## Highlights

• The diversity of the human T cell repertoire is known to reduce in old age.
• Canine T cell receptor repertoire diversity was analysed using CDR3 spectratyping.
• An age-associated disturbance was identified in the T cell repertoire of dogs.

## 1 Introduction

Involution of the thymus leads to a reduction in the output of naive T cells for recruitment into the peripheral circulation and secondary lymphoid tissues (Lynch et al., 2009). Consequently, with increasing age, homeostatic maintenance of the peripheral T cell pool increasingly relies on proliferation of existing naive and memory T cells (Kilpatrick et al., 2008, Naylor et al., 2005). This can lead to diminished diversity of the T cell repertoire with the result that geriatric individuals can have a reduced capacity to mount an effective immune response to novel antigenic challenge (Naylor et al., 2005), such as during infection (Messaoudi et al., 2004) and following vaccination (Saurwein-Teissl et al., 2002, Yager et al., 2008). This is typically seen in geriatric humans receiving seasonal influenza vaccines, where the efficacy is reduced (Goodwin et al., 2006, Hannoun et al., 2004). Additionally in old age, expansion of the memory T cell compartment has been found to occur more rapidly in CD8^+^ compared to CD4^+^ cells (Blackman and Woodland, 2011, Goronzy et al., 2007). In humans, this is believed to be the result of instabilities in homeostatic maintenance (Czesnikiewicz-Guzik et al., 2008), and the clonal expansion of specific memory CD8^+^ T cells due to antigenic stimulation by persistent viral infection, such as with cytomegalovirus (CMV) (Olsson et al., 2000, Pawelec et al., 2005). Which leads to further reductions in the diversity of the T cell repertoire in old age.

The diversity of the T cell repertoire is determined during lymphocyte development in the thymus through the rearrangement of V, D, J and C gene segments to form the alpha and beta chains of the T cell receptor (TCR). In dogs, the *TCRB* locus is composed of 35–37 V gene segments located upstream of two D-J-C clusters, each containing one D, six J and one C gene segments (Mineccia et al., 2012). This is similar to the structure of the *TCRB* locus in humans and mice, except that they have 64 and 34 V gene segments respectively (Glusman et al., 2001). The *TCRA* locus, which in humans consists of 56 V gene segments followed by 60 J genes and a single C gene segment (Glusman et al., 2001), has yet to be fully characterised in the dog genome. Imprecise joining of the V, D and J gene segments, due to asymmetric opening of the hairpins on the ends of the coding sequences, and the addition and removal of nucleotides by the actions of terminal deoxynucleotidyl transferase (TdT) and endonuclease, contributes further to the diversity of the TCR chains (Gellert, 2002).

The peripheral T cell repertoire can be evaluated by analysing variation in the sizes of the CDR3 segment in mRNA from blood samples and tissues (Cochet et al., 1992). CDR3 spectratyping is an established method which has been used in a variety of species, including humans (Gorski et al., 1994), mice (Cochet et al., 1992, Pannetier et al., 1993), primates (Chen et al., 1993) and pigs (Baron et al., 2001). In dogs, CDR3 spectratyping of *TCRB* families has previously been used to examine changes in the T cell repertoire of dogs with X-linked severe combined immunodeficiency following bone marrow transplants (Vernau et al., 2007). However, this latter study was undertaken prior to robust annotation of the canine genome at the *TCRB* locus, and therefore only examined a limited number of *TCRB* V gene families. In humans and laboratory rodents, CDR3 spectratyping has been used to identify age associated clonal expansions/deletions in the T cell repertoire, which cause the CDR3 lengths within a particular *TCRB* V gene family to skew away from a normal or “Gaussian” distribution (Mosley et al., 1998, Wack et al., 1998). CDR3 spectratypes from mice aged 16 or 24 months were frequently distorted, compared to spectratypes from 8-month-old mice, which were typically symmetrical (Mosley et al., 1998). While in humans, clonal expansions in *TCRB* V gene families were more frequently observed in individuals over 65 years of age, compared to young adults (Wack et al., 1998).

The aim of the present study was to develop a CDR3 spectratyping assay that would allow analysis of the *TCRB* V gene families expressed in canine blood samples to test the hypothesis that there is a more restricted T cell repertoire in geriatric, compared with young dogs.

## 2 Materials and methods

### 2.1 Sample population

EDTA blood samples were obtained from 32 Labrador retriever dogs, referred to the Queen Mother Hospital for Animals at the Royal Veterinary College, following completion of diagnostic testing, with ethical approval (approval number URN2016/1475) and with informed owner consent for use of these samples for clinical research. The study population consisted of 16 young dogs, aged between 0 and 3 years, and 16 older dogs, aged between 10 and 13 years. All the samples were from dogs with a lymphocyte count within the reference interval (1.0–4.8 × 10^9^/L), and where possible a normal haematology result (5.5–8.5 × 10^12^/L red blood cells, 6.0–17.1 × 10^9^/L white blood cells, 3.0–11.5 × 10^9^/L neutrophils, 0.15–1.5 × 10^9^/L monocytes), as determined by an automated haematology analyser (ADVIA 2120i, Siemens Healthcare, Camberley, UK).

### 2.2 Nucleic acid extraction

RNA was purified from EDTA blood using the PureLink™ Total RNA Blood Purification Kit (Promega, Southampton, UK), according to the manufacturer's instructions. An on-column digestion step (RNase free DNase set, Qiagen, Manchester, UK) was performed to remove contaminant genomic DNA. RNA quality and quantity were assessed using the 2100 Bioanalyzer system (Agilent Technologies, Stockport, UK), where samples with a RIN ≥ 6 were considered of a suitable quality to proceed to complementary DNA synthesis. Reverse transcription of mRNA to complementary DNA (cDNA) was performed using an Oligo(dT)_15_VN primer (Sigma-Aldrich, Poole, UK) and ImmProm-II™ reverse transcriptase (Promega).

### 2.3 Primer design

Sense primers located in 15 canine *TCRB* variable (V) gene regions (Sigma-Aldrich) were designed based on previous characterisation of this region of the dog genome (Matiasovic et al., 2009, Mineccia et al., 2012) and sequence information available in the IMGT Repertoire on the IMGT^®^, international ImMunoGeneTics information system^®^, website (http://www.imgt.org) (Lefranc et al., 1999) (Table 1). Each sense primer was designed to be used in combination with a common antisense primer, located in the *TCRB* constant (C) region, which was labelled with a fluorescent WellRED™ dye (D4-PA) (Sigma-Aldrich) (Table 1). Primer specificity was confirmed by cloning and sequencing PCR products generated using cDNA from thymus tissue.

**Table 1:** Primers used to amplify canine *TCRB* gene segments.

*TCRB* gene	Primer Sequence (5′–3′)	Source
V1	CCCAGTACCCTTGGATGAGC	Novel
V3.2	CCTCAAGCAACTGCTGAACGTT	Novel
V4.4	CACCGAAGCTCATGTTTGCC	Novel
V5.2	CCCTGAGATGTTCCCTTATCTCT	Matiasovic et al. (2009)
V5.4	ATCTCGTCTCTGGACACAACACT	Matiasovic et al. (2009)
V7	GGCCCGGAGTTTCTGGTTTA	Novel
V10	ATGGGCCGAGGCTGATCTAT	Novel
V16	ATTTCTTTCCAGGATAACGCTGT	Matiasovic et al. (2009)
V18	CCGAAGACGGACTTAGCATC	Matiasovic et al. (2009)
V20	CCTTACCCTTATGGTGACCTCTA	Matiasovic et al. (2009)
V24	CTACGGCTGATCTCCTACTCCTT	Matiasovic et al. (2009)
V25	CGACAAGACCCAGGAAAGGC	Novel
V26	AATCTTTTCCCCTGACCCTGG	Novel
V28	AAGAAGGATGCCTTCCCCTTG	Novel
V29	TACCCAAGTCACCTTGATGT	Matiasovic et al. (2009)
Constant	(D4-PA)TCTGATGGTTCAAACACTGTGAC	Novel

### 2.4 Generation of amplicons for CDR3 spectratyping

Polymerase chain reaction (PCR) was used to amplify individual *TCRB* V genes for CDR3 spectratyping. Each 25 μl reaction contained 2 μl sense and antisense primer (2.5 pmol/μl), 2 μl cDNA as the template, 5 μl MyTaq™ Reaction Buffer, and 0.2 μl MyTaq™ HS DNA Polymerase (Bioline, London, UK). Reactions were heated to 95 °C for 3 min followed by 37 cycles of 95 °C for 15 s, 60 °C for 20 s and 72 °C for 30 s; with a final extension step of 72 °C for 10 min, using a G Storm thermocycler (Gene Technologies Ltd, Essex, UK). Where possible, reactions were multiplexed to reduce the total number of reactions required for each dog. For reactions where two *TCRB* V genes were multiplexed in the same tube the elongation step was extended to 90 s.

### 2.5 Analysis of CDR3 spectratypes by capillary electrophoresis

After amplification, 0.5 μl of PCR product was added to 39.5 μl sample loading solution along with 0.5 μl DNA size standard 400 (Beckman Coulter Ltd, High Wycombe, UK). The fluorescently labelled amplicons were separated on a GenomeLab™ GeXP Genetic Analyser (Beckman Coulter) by capillary electrophoresis. After separation, the peaks were initially analysed in the fragment analysis module of the GeXP system software, where the peak sizes (bp) and heights (rfu) are reported as a spectragram.

Peak area data, generated from the spectragrams, was used to calculate relative fluorescence intensity (RI) for each peak, where %RI = 100 × each clonal peak area/the total peak area for that *TCRB* V gene. Based on a previously published scheme (Ma et al., 2011), spectragrams were assessed for evidence of clonal expansion/deletion by virtue of a) the presence of a single peak at any position in the spectragram with an RI > 35%, b) the presence of two peaks at any position in the spectragram with an RI > 25%, or c) the presence of a dominant peak with an RI > 25% in a non-central position in the spectragram. The number of clonal peaks present in each spectragram was also used to calculate the total number of peaks for each dog, to give an estimate of clonal diversity in the T cell repertoire.

## 3 Results

**Fig. 1:**
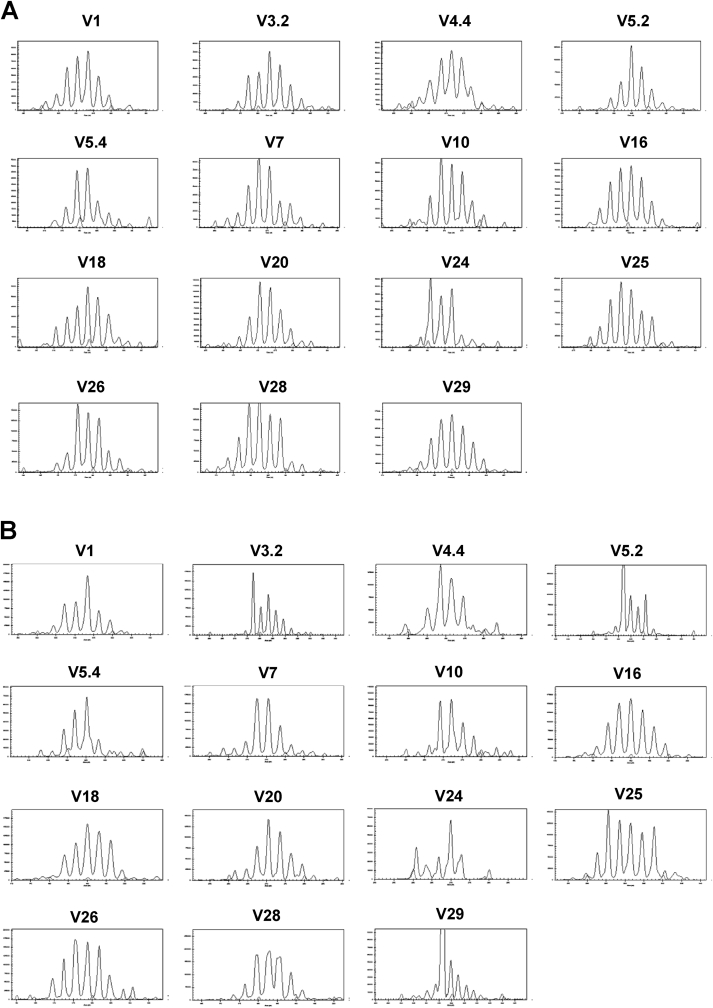
**Examples of CDR3 spectratypes in (A) a young and (B) an old Labrador retriever.***TCRB* amplicons for 15 different variable (V) gene families were generated from peripheral blood cDNA using specific sense primers, in combination with a common antisense primer designed to anneal to the constant (C) region. These were separated by capillary electrophoresis and the size and fluorescence intensity of the separated products for each TCRB V gene are shown as peaks on each spectragram.

**Fig. 2:**
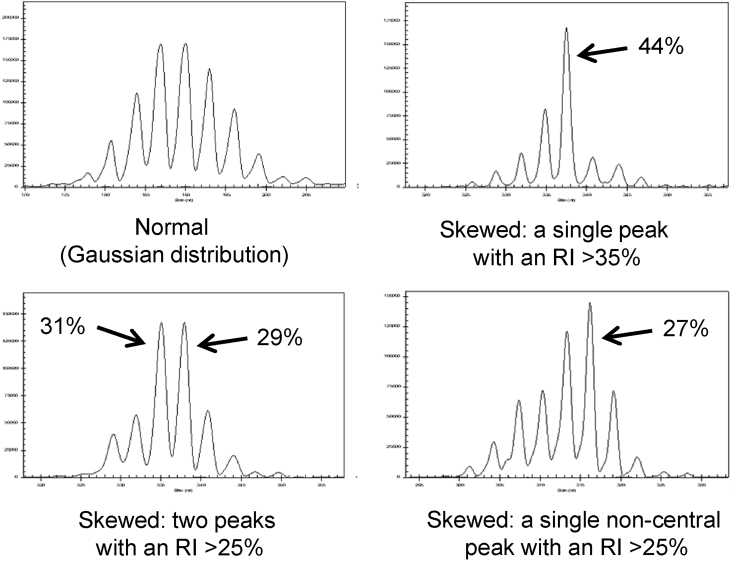
**Assessment of CDR3 spectratypes for the presence of a skewed distribution.** A relative fluorescence intensity value was calculated for each peak (%RI = 100 × each clonal peak area/the total peak area for that TCRB V gene) and used to examine the spectratypes for evidence of a skewed distribution, suggestive of the presence of clonal expansion/deletion. Representative spectragrams are shown for each distribution category.

The expression patterns of 15 *TCRB* V gene families were evaluated in peripheral blood samples from 16 young (0–3 years) and 16 old (10–13 years) Labrador retriever dogs. Examples of the CDR3 spectragrams generated are shown in Fig. 1. Spectragrams were assessed, based on objective criteria (Ma et al., 2011), to determine whether they demonstrated a Gaussian distribution of peaks, or alternatively showed evidence of a skewed distribution. Examples of different distribution categories are shown in Fig. 2.

**Fig. 3:**
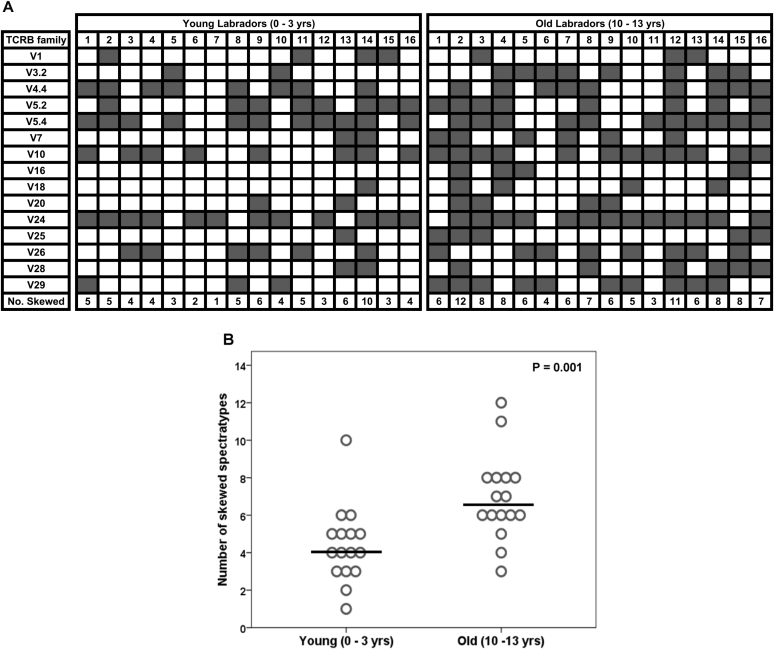
**The number of CDR3 spectratypes with a skewed distribution is increased in old Labrador retrievers.** (A) Matrix showing the distribution of normal (white) and skewed (dark grey) spectratypes in young and old Labrador retrievers. (B) Dot plot showing the number of skewed spectratypes identified in young and old Labrador retrievers. Each dog is represented by a circle within their age group. A trend line (in black) is positioned at the median of each age group. The P value was calculated using a Mann Whitney *U* test comparing the two groups.

The Labrador retrievers were assessed for the number of skewed spectratypes present in their TCR repertoire (Fig. 3A). All of the dogs demonstrated at least one skewed spectratype, but the older Labrador retrievers were found to have significantly more skewed spectratypes compared with the young dogs (P = 0.001) (Fig. 3B). This suggests that there is an age-associated increase in the presence of clonal expansion/deletion within the TCR repertoire in Labrador retrievers. *TCRB* V5.4 and V24 were identified to be the V gene families most likely to produce a skewed spectratype in this study population. In four of the Labrador retrievers (2 young and 2 old) it was not possible to generate a PCR product for one of the *TCRB* V genes (either *TCRB* V3.2 or V24), despite repeated attempts alongside a positive control, which was also considered to represent a skewed distribution (i.e. V family missing from the repertoire).

**Fig. 4:**
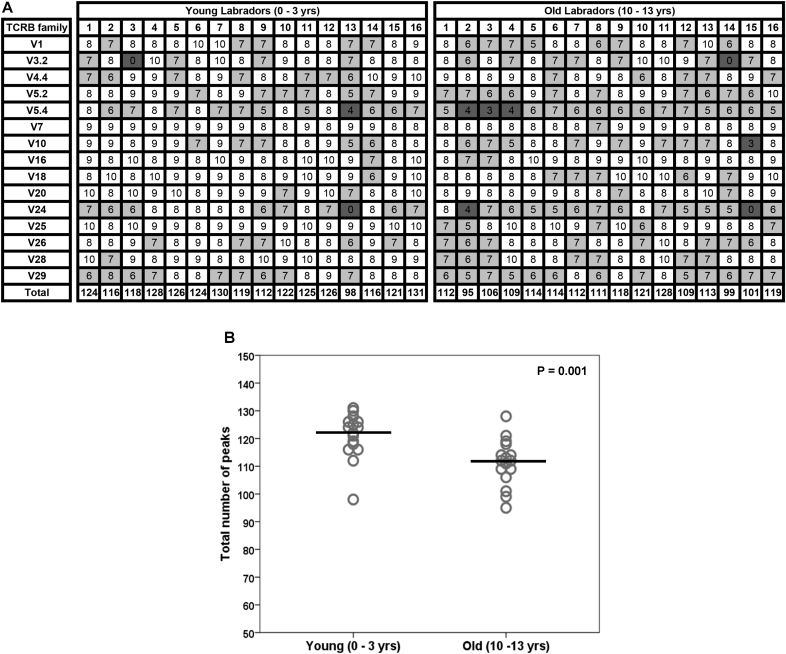
**The number of clonal peaks in CDR3 spectratypes is reduced in old Labrador retrievers.** (A) Matrix showing the number of peaks in individual CDR3 size spectratypes. Spectratypes with 8–10 peaks are shown in white, those with 5–7 peaks are in light grey, and those with ≤4 are shown in dark grey. (B) Dot plot showing the total number of peaks across all the CDR3 size spectratypes for each individual dog. Each dog is represented by a circle within their age group. A trend line (in black) is positioned at the median of each age group. The P value was calculated using a Mann Whitney *U* test comparing the two groups.

The CDR3 spectratypes were analysed further by examining the number of clonal peaks generated for each of the *TCRB* V families (Fig. 4A). The spectratypes were found to contain between 3 and 10 peaks, suggesting the number of individual clones differed for each *TCRB* V family and between individual dogs. Spectratypes with a Gaussian distribution would be expected to contain 8-10 peaks (Gorski et al., 1994, Pannetier et al., 1993). In the young dogs, 73% of the spectratypes contained 8–10 peaks, while in older dogs this was only 53%, suggesting that the number of spectratypes with a normal distribution has reduced with age. The *TCRB* V families most likely to have a reduced number of peaks in their spectratypes were *TCRB* V5.4 and V24, although few had reduced to the point where they were considered to be oligoclonal. The total number of peaks for each dog was then calculated to examine the influence of age on the overall clonal diversity of the T cell repertoire (Fig. 4B). There was a significant reduction in the total number of clonal peaks in the older Labrador retriever group compared with the younger group (P = 0.001). This suggests that in the older dogs there is reduction in the overall clonal diversity of their TCR repertoire.

## 4 Discussion

This study investigated age related perturbations in the peripheral T cell repertoire by examining *TCRB* V family diversity in peripheral blood of dogs using CDR3 size spectratyping. This revealed a significant increase in the number of *TCRB* V gene families showing a skewed distribution in their spectragram profile in the old dogs compared with the young dogs. There was also a significant reduction in the number of clonal peaks present in the spectratypes of old dogs, compared with those of the young dogs. These research findings suggests that there is an age-associated perturbation in the diversity of the canine T cell repertoire.

Previous publications analysing T cell repertoires in humans (Gorski et al., 1994) and laboratory rodent models (Pannetier et al., 1993) have reported that healthy individuals demonstrate CDR3 spectratypes that are usually composed of 8–10 peaks of different sizes, separated by ∼3 bp, with a Gaussian-like distribution. In the present study the CDR3 spectratypes were assessed for divergence away from this Gaussian distribution based on a set of objective criteria (Ma et al., 2011). In the Labrador retriever dogs assessed, the number of CDR3 spectratypes with a skewed distribution was found to be significantly increased in the old dogs compared with the young dogs, suggesting that with advancing age there is evidence of clonal expansion/deletion in the T cell repertoires of some dogs. In four of the dogs (2 young and 2 old) the lack of a PCR product for either V3.2 or V24 suggests these dogs might be missing a *TCRB* V gene family from their T cell repertoire. Alternatively, polymorphisms in the region of primer binding might lead to the lack of a PCR product. The complete loss of a *TCRB* V gene family from the anticipated repertoire is likely be the result of positive or negative selection during T cell development in the thymus. However, analysis of both germline and mRNA sequences would be required to confirm this is occurring.

The majority of CDR3 size spectratypes in both young and old Labrador retrievers were found to contain 8-10 peaks, as would be expected in a diverse repertoire (Gorski et al., 1994, Pannetier et al., 1993). However, in some of the spectratypes, the number of clonal peaks had reduced to give a more oligoclonal profile containing 3–4 peaks, suggesting that in some dogs there had been a reduction in the clonal diversity of certain *TCRB* V gene families. When the total number of CDR3 peaks was analysed, there was a significant reduction in polyclonality in the old dogs compared with young dogs. Taken together these research findings indicate that the overall diversity of the T cell repertoire in the older dogs was reduced, compared with the young dogs. However, since over 50% of the CDR3 spectratypes in the older Labrador retrievers were found to still have a normal polyclonal profile, this suggests that the diversity of the canine T cell repertoire in old age is maintained to some extent, and that some *TCRB* V gene families become more restricted than others with increasing age in this particular breed.

CDR3 size spectratyping in humans (Schwab et al., 1997, Wack et al., 1998) and laboratory rodents (Messaoudi et al., 2004, Mosley et al., 1998) has shown that age related changes in the T cell repertoire occur in both CD4^+^ and CD8^+^ T cells. However, changes to the CD8^+^ population have been found to occur earlier and are more pronounced than is apparent in the CD4^+^ population (Wack et al., 1998). Due to the limited volume of blood available for the dogs in this study, isolation of CD4^+^ and CD8^+^ T cells was not feasible, therefore the age-related changes observed represent a mixture of the changes occurring to both the CD4^+^ and CD8^+^ populations. Additionally, both naïve and memory T cell populations will have contributed to the RNA extracted from the canine blood samples. We have previously demonstrated an age-associated decline in thymic output in dogs (Holder et al., 2016), which would imply that dogs at varying ages are likely to exhibit altered proportions of naïve and memory T cells. However, identifying age-associated restrictions in the repertoires of naïve and memory populations was not possible with the limited volumes of blood available.

In humans, repertoire constriction in the CD8^+^ memory T cell compartment is characterised by the presence of large numbers of clonally expanded T cells (Blackman and Woodland, 2011), which is thought to be driven by chronic antigenic stimulation, typically due to persistent viral infection, such as with cytomegalovirus (Hadrup et al., 2006, Khan et al., 2002). Few of the dogs in this study produced CDR3 spectratypes with an oligoclonal profile, and none were found to be monoclonal in appearance, suggesting that perhaps the T cell repertoire in dogs is not driven by latent viral infections to the same extent as is seen in some humans. There is little evidence of persistent beta herpes virus infection equivalent to CMV in the dog population. Canine herpes virus 1, an alpha herpes virus, is known to cause a fatal haemorrhagic disease in puppies and disorders of the reproductive system in adult dogs, as well as sometimes being associated with respiratory and ocular disease (Evermann et al., 2011). However, the presence of latent alpha herpes viruses in humans do not appear to have the same influence as CMV on the aging immune system (Fulop et al., 2013), possible because they target mucosal and neuronal cells rather than lymphoid cells. Alternatively, the presence of oligoclonal and monoclonal expansions in the CD8^+^ memory compartment might be masked by the mixed nature of the T cell populations used for the current CDR3 spectratyping assay.

Individual variation in the number of *TCRB* V families demonstrating a non-Gaussian distribution was observed in dogs from the same age group. Similar variation is also reported between individuals in human (Schwab et al., 1997) and mouse (Mosley et al., 1998) studies. However, the number of young Labrador retrievers demonstrating skewed CDR3 profiles appears to be higher than might be expected, with all the young dogs having at least one skewed CDR3 size spectratype and more than 50% of dogs having four or more skewed spectratypes. Previous studies in humans and mice have not identified a substantial degree of perturbation in the T cell repertoires of younger subjects, where restricted *TCRB* V profiles were only found in two of 14 young adults aged 22–34 years (Schwab et al., 1997), and <10% of spectratypes in 8 month old mice had a skewed profile (Mosley et al., 1998). The samples in the present study came from dogs undergoing treatment at a tertiary-care veterinary hospital. Although they were selected on the basis of being 'haematologically healthy' it is possible that their disease processes contributed to some of the changes observed in the CDR3 size spectratypes, or that restrictions in their T cell repertoire have influenced their susceptibility to disease.

Samples from human clinical patients have been found to have an increased prevalence of CDR3 size spectratypes demonstrating oligoclonal profiles, or polyclonal profiles which contain expanded clones, compared to those from healthy individuals (Pannetier et al., 1995). It is interesting to note that those dogs with the greatest number of skewed CDR3 size spectratypes were diagnosed with cancer, atopic dermatitis and pancreatitis, which evidence from the current literature suggests might be associated with perturbations in the immune system. Studies in human blood and tissue samples from patients with melanoma and colorectal cancer have identified greater restrictions in the *TCRB* repertoire, compared to healthy controls (Degauque et al., 2004, Ochsenreither et al., 2010). In a mouse model of atopic dermatitis, oligoclonal expansions of *TCRB* V2, V4 and V6 genes were associated with the development of skin lesions (Matsuoka et al., 2005). While, a recent genome-wide association study (GWAS) performed in dogs has identified polymorphisms in the *TCRB* locus that are significantly associated with chronic pancreatitis (Dr P. J. Watson, University of Cambridge, personal communication). To establish that differences observed between the young and old Labrador retrievers are entirely due to age, a clinically normal population of dogs would have been required for this study. However, the logistical, ethical and legislative issues that surround the taking of blood from healthy dogs in the UK meant this was not feasible.

Some of the changes observed in the distribution of peaks on CDR3 size spectratypes might represent a temporary increase in certain T cell clones due to recent infection or vaccination. A longitudinal study following the effects of lymphocytic choriomeningitis virus infection in mice identified clonal expansions in the T cell repertoire which occurred during the acute phase of infection, between days 6 and 12, but which had disappeared at day 42 of the study, following clearance of the virus (Lin and Welsh, 1998). Similar disturbances in the T cell repertoire are observed in patients with HIV infection, some of which were found to resolve following antiretroviral therapy (Gea-Banacloche et al., 2000). Vaccination has also been shown to cause the expansion of specific T cell clones, as demonstrated in a human study following the response to hepatitis B vaccine, which identified a monoclonal expansion in CD8^+^ T cells expressing *TCRB* V3 (Hohn et al., 2002). To establish whether this could explain some of the skewed CDR3 size spectratypes present in the Labrador retrievers used for this study, longitudinal samples would have been required, preferably pre- and post-vaccination.

## 5 Conclusion

Development of a canine CDR3 size spectratyping assay has identified age-related perturbations in the TCR repertoire of Labrador retriever dogs. CDR3 size spectratyping could be used to examine the influence of T cell repertoire diversity on age and breed related differences in susceptibility to infectious, immune-mediated and neoplastic disease, and response to vaccination. Thus, there is an opportunity to gain insight into immune-competence while investigating immunosenescence and immune dysfunction in an important veterinary species that also acts as a model for human aging and disease.

## Conflict of interest statement

The authors declare that they have no competing interests.

## Appendix A Supplementary data

Data profile Data profile
